# Identification of genomic drivers for the therapeutic response of Cabozantinib in patients with metastatic renal cell carcinoma

**DOI:** 10.1007/s00345-024-04783-y

**Published:** 2024-02-22

**Authors:** Angelika Borkowetz, Ulrich Sommer, Gustavo Baretton, Carsten Gruellich, Björn Thorben Bürk, Holger H. H. Erb, Christian Thomas, MORECAB Consortium

**Affiliations:** 1 Department of Urology, University Hospital Carl Gustav Carus, Technische Universität Dresden, Fetscherstraße 74, 01307 Dresden, Germany; 2 German Cancer Consortium (DKTK), Site, Dresden, Germany; 3 Institute of Pathology, University Hospital, Technische Universität Dresden, Dresden, Germany

**Keywords:** Cabozantinib, Genomic alterations, Metastatic renal cell carcinoma

## Abstract

**Purpose:**

Cabozantinib (CAB) as monotherapy or in combination with immune checkpoint inhibitors is used for systemic treatment of metastatic renal cell carcinoma (mRCC). However, little is known about predictors of treatment response to CAB. For this reason, known genomic drivers were examined to identify potential predictors of treatment response with CAB.

**Methods:**

Twenty mRCC patients receiving monotherapy (≥ first-line) with CAB were prospectively included. DNA was extracted from archived primary tumors or metastatic tissue. Targeted DNA sequencing was performed using a gene panel including 328 genes (QIAseq Targeted DNA V3 Panel, Qiagen). The variant evaluation was performed using Varsome. The endpoints were treatment-failure-free-survival (TFFS) to CAB.

**Results:**

26% of patients received systemic RCC treatment as the primary option. Six patients were treated with CAB in first-line (1L) and 12 patients in ≥ 2L. The median follow-up after initiation of systemic treatment was 26.7 months (mo). The *PBRM1* (7 alleles), *SETD2* (7 alleles), *VHL* (11 alleles), and *CHEK2* (14 alleles) genes were most frequently altered. The median time to TFFS was 10.5 mo (95% confidence interval (CI) 6.2–14.7 mo). There was a longer treatment response to CAB in patients with alterations of the *SETD2* gene (*SETD2* alteration median TFFS not reached vs. no *SETD2* alterations 8.4 mo (95% CI 5.2–11.6 mo); *p* = 0.024).

**Conclusion:**

Pathogenic variant genes may indicate treatment response to systemic therapy in mRCC. Patients with alterations of the *SETD2* gene show longer responses to CAB treatment.

**Supplementary Information:**

The online version contains supplementary material available at 10.1007/s00345-024-04783-y.

## Introduction

Renal cell carcinoma (RCC) represents 5% and 3% of all cancers in men and women, respectively [1].

While the primary treatment option for localized or oligometastatic RCC is surgery, multiple metastasized RCC (mRCC) is typically treated by systemic treatment. Herein, tyrosine kinase inhibitors (TKI), mTOR inhibitors, and immune checkpoint (ICP) inhibitors or a combination of them play the main role [2]. In first-line (1L) treatment, depending on the International Metastatic Renal Cell Carcinoma Database Consortium (IMDC) risk group, the combination of ICP/TKI or ICP/ICP or the monotherapy with Cabozantinib (CAB) is recommended [2].

The TKI CAB acts across vascular endothelial growth factor (*VEGF*) receptors, rearranged during transfection (*RET*), mesenchymal–epithelial transition factor (*MET*), and *AXL* pathway focusing on impairment of neovascularization pathways in mRCC [3]. When used as 1L monotherapy or combined with the ICP Nivolumab, the survival rates are significantly better than TKI Sunitinib [4, 5].

The efficacy of CAB monotherapy was demonstrated in 2L treatment after *VEGF* inhibitors in the Phase 3 METEOR study [6] or in 1L treatment in the Phase II CABOSUN trial against Sunitinib in patients with intermediate and poor risk mRCC [4]. The CONTACT-03 trial presented no benefit for the combination of CAB and Atezolizumab compared to CAB monotherapy after progression with ICP in 1L treatment. However, a median progression-free survival of 10.8 months (mo) for the CAB monotherapy arm was reported [7]. However, the CABOSEQ trial demonstrated that 2L CAB still showed meaningful efficacy independent from the 1L treatment [8]. The CaboPoint trial investigating 2L CAB after ICP showed a 31.7% objective response rate of CAB after ICP/ICP and 25% after ICP/TKI [9].

Although the Von Hippel-Lindau (*VHL*) tumor suppressor is the most frequently detected mutation in ccRCC associated with the development and progression of RCC, little is known about genomic alterations and their impact on the therapeutic response of different treatment agents [10]. It has been shown that the altered gene of the tumor suppressor *PBRM1*, which represents the second most common mutation in RCC (40%), is associated with longer progression-free survival and better treatment response to Sunitinib [11]. So far, there is no data on identified genomic alterations that might impact CAB response.

Due to tumor heterogeneity and clonal evolution under systemic treatment, all mRCCs become inevitably refractory. Biomarkers predicting survival and favoring one approved substance over the others due to their target spectrum are currently unavailable but are urgently needed for tailoring an individualized treatment.

This study aimed to identify a genomic pattern predicting treatment response in 20 mRCC patients.

## Methods

The MORECAB trial is a sub-study of the Molecular Determinants for Therapy Response on Renal Cell Carcinoma (MORE) trial investigating molecular biomarkers allowing prediction of disease progression on any treatment option in advanced or mRCC (clinical trials registry *NCT02208128).* In this extension, the MORECAB trial analyzed genomic alterations as molecular biomarkers for disease progression under treatment with CAB in any treatment line. The use of archived material was approved by the Ethics Committee of the University of Heidelberg (Study no. S-539/20139).

Patients receiving CAB monotherapy in all treatment lines for advanced or metastatic RCC were prospectively investigated. All entities of RCC (clear cell, chromophobe, papillary) could be included. In the case of ≥ 2L treatment with CAB, previous treatment with TKI, mTOR inhibitors, or ICP was allowed. Treatment with CAB could have already started before the patient was included in the study. After consenting to the study, archived primary or metastatic tumor tissue was subjected to central review. Treatment was monitored and data were collected by the study center at the Department of Urology at the Technische Universität Dresden. Imaging Computer tomography (CT) of the chest and CT or magnetic resonance imaging of the abdomen) was performed every 3 mo.

Histopathological, clinical, and treatment data were collected prospectively in a single-center approach. Survival data from the time of study participation were prospectively recorded. Overall (OS), cancer-specific (CSS), treatment-failure-free survival (TFFS) data, and the best response under treatment were investigated.

Paraffin sections of tissue were selected by secondary pathological review (US). Paraffin sections were cut into 2 µm sections and stained with hematoxylin–eosin. Tumor and tumor-free regions were marked.

Genomic alterations were analyzed by targeted DNA sequencing. According to the manufacturer’s instructions, the DNA of formalin-fixed paraffin-embedded (FFPE) tumor tissue was extracted using the QIAamp DNA FFPE Tissue Kit (QIAGEN, Hilden, Germany).

In total, 250 ng of DNA was used as input for library preparation. Extracted DNA was amplified using the Human Comprehensive Cancer Panel (QIAseq Targeted DNA V3 Panel; QIAGEN, Hilden, Germany) according to the protocol "QIAseq Targeted DNA V3 Panel, (QIAGEN, Hilden, Germany). This panel includes 328 genes commonly mutated in cancer. All steps of library preparation were performed according to the manufacturer’s protocol. During the library preparation, unique molecular barcodes and sample-specific indices were incorporated according to the protocol. Indexed libraries were paired-end (2 × 150 bp) on an Illumina NextSeq platform (Illumina, San Diego, CA, USA). HG19 was used as a genome reference for bioinformatic analyses performed using the Biomedical Workbench from CLC (12.0.3) using a customized analysis algorithm with the following filters: coverage ≥ 100, allele frequency ≥ 5%. In addition, a variant evaluation was performed by Varsome (Varsome, Saphetor SA, Lausanne, Switzerland; 31.12.2022). Only variants presented in the tumor areas but not in the tumor-free areas were included in the final variant list.

SPSS Statistics v29.0 (IBM, Armonk, NY, USA) was used for statistical analyses. Differences between groups were analyzed using the Chi^2^ test or Student’s t-test. We formulated a two-sided hypothesis to explore gene alterations regarding treatment response to CAB. Kaplan–Meier estimate has been used for survival analysis. Cox regression analyses were performed to identify predictors for OS, CCS, or TFFS for CAB. *P*-values of ≤ 0.05 were considered as statistically significant. Only pathogenic and likely pathogenic gene alterations were investigated in survival analysis. For oncoplots description, R v4.1.2 (R Foundation for Statistical Computing, Vienna, Austria) and Maftools were used for the analysis and plotting [12].

## Results

20 patients with advanced or mRCC were included. Patient’s characteristics are depicted in Suppl. Table 1. One patient who lost to follow-up and one patient who committed suicide immediately after the initiation of CAB treatment were not included in survival but in genomic analysis. In 19 patients, primary tumor tissue was analyzed. In one patient, only metastatic tumor tissue was available. The median follow-up was 26.7 mo and the median follow-up from the initiation of CAB to the last follow-up was 21.26 mo. 17/20 patients died during the study. Death was cancer specific in 12 patients (71%).

**Fig. 1:**
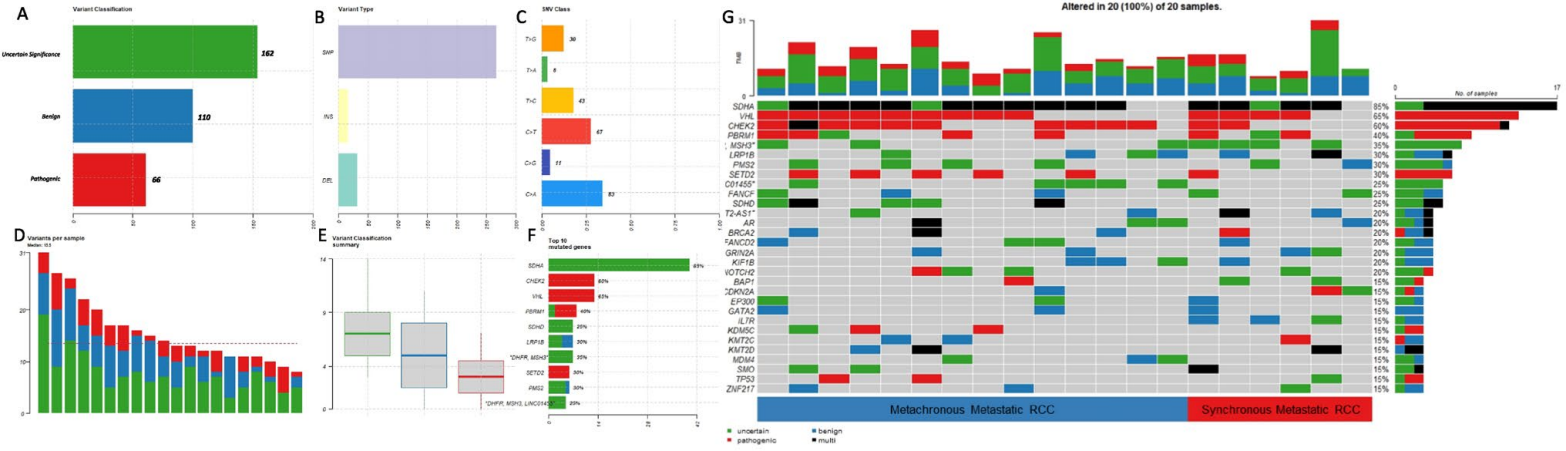
1 **A** Variant classification, 1 **B** Variant types, 1 **C** Classification of single nucleotide variants, 1 **D**) number of variants per patient, 1 **E** Summary of variants classifications, 1 **F** most altered genes, 1**G** oncoblot depicting the type of gene alterations per patient and stratified to metastatic pattern

In the variant assessment of genomic alterations (Suppl. Table 2), 16 pathogenic gene alterations (on 39 alleles), 10 likely pathogenic gene alterations (on 27 alleles), 70 gene alterations with uncertain significance (on 162 alleles), 23 likely benign gene alterations (on 31 alleles), and 48 benign gene alterations (on 79 alleles) were described. The most common pathogenic/likely pathogenic gene alterations were *PBRM1* (7 alleles), *SETD2* (7 alleles), *VHL* (11 alleles), and *CHEK2* (14 alleles). *BRCA2* alteration was found on 2 alleles. Missense mutations were the most common, followed by frame-shift deletions and non-sense mutations (Fig. 1A). Single nucleotide polymorphisms were the most common mutation type (Fig. 1B**)**. In this cohort, there are no obvious differences in the mutation’s characteristics in patients with metachronous and synchronous mRCC. (Fig. 1G).

Median CSS was 5.9 years (95%-CI 2.6–9.3 years). The median OS starting from 1L treatment was 24 mo (95%-CI 19.8–35.4 mo) and the median OS from the beginning of CAB was 21.6 mo (95%-CI 18.2–24.4 mo). None of the proven pathogenic/likely pathogenic gene alterations were associated with OS.

**Fig. 2:**
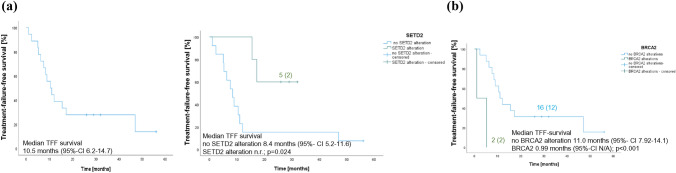
TFFS of Cabozantinib in all patients (**A**), TFFS of Cabozantinib in patients with (and without SETD2 alterations (**B**), TFFS of Cabozantinib in patients with BRCA2 gene alterations and no BRCA2 gene alteration

The median TFFS with any line CAB was 10.5 mo (95%-CI 6.2–14.7 mo) (Fig. 2A**, **Suppl. Table 3). The TFFS was longer in the presence of *SETD2* alterations (*SETD2* alteration median TFFS not reached vs. no *SETD2* alterations 8.4 mo (95%-CI 5.2–11.6 mo); *p* = 0.024) (Fig. 2B). Moreover, in the case of proven *BRCA2* alterations, the TFFS was shorter (no BRCA2 alteration median TFFS 11 mo (95%-CI 7.92–14.1 mo) vs. *BRCA2* alterations 0.99mo (95%-CI N/A); *p* < 0.001) (Fig. 2C). Neither pathogenic/likely pathogenic gene alterations (*VHL*, *PBRM2*, *mTOR*, *BAP1*, *EAPS*, *CHEK2*) nor their combination influenced TFFS.

**Fig. 3:**
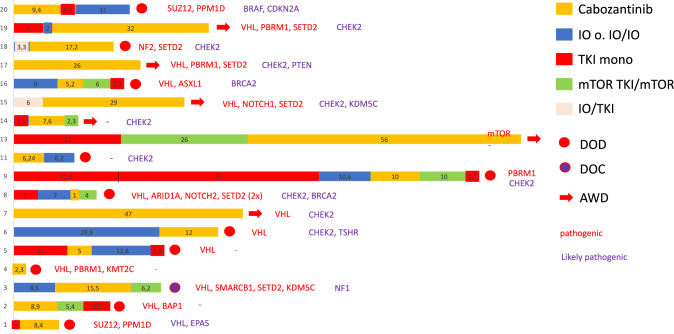
Swimmer plot for treatment sequences and presented pathogenic variant genes (*n* = 18)

The swimmer plot (Fig. 3) displays the treatment sequence (*n* = 18). 8 patients (44%) were treated by CAB ≥ 12 mo, whereas three patients (17%) were still under treatment at the last investigation. Those patients presented a VHL alteration (*n* = 6; 33%). Two patients presented a *PBRM1* (11%), and another five (27%) had *SETD2* alterations. Six patients (33%) presented a *CHEK2* alteration.

## Discussion

In this exploratory analysis, we investigated patients with mRCC treated by CAB in different treatment lines.

The CABOREAL study investigating CAB in ≥ 2L treatment showed a median OS of 14.4mo and a 12-month OS rate of 56.5% [9]. Data from the open-label phase II CaboPoint trial revealed that there is still a high objective response rate in 2L treatment with CAB [9]. Gan et al. showed that CAB in higher treatment lines still showed good response rates with ORR of 26–29% in 2L to 4L. After 1L IO treatment, CAB treatment resulted in an ORR of 22%, TFFS of 5.4 mo, and a median OS of 17.4 mo [13]. We could also demonstrate a prolonged median OS of 21 mo starting from the beginning of CAB in a cohort with ≥ 2L CAB. Vano et al. demonstrated that ≥ 2L sequence Nivolumab and then CAB were superior to the ≥ 2L sequence CAB and then Nivolumab [14]. However, CAB seems to be an effective TKI in higher treatment lines, especially after ICP/ICP frontline treatment.

Although ICP is used in the therapy of mRCC, PD1/PDL1 status does not guide treatment decisions. In the METEOR and CABOSUN cohort, tumor cell PDL1 expression was associated with poorer PFS and OS. However, it was not predictive of treatment response for CAB [15]. AXL/GA6 scoring seems to predict treatment response in CAB therapy [16]. However, biomarkers have not been included in treatment decisions so far.

The most common gene alterations in the primary tumor in RCC are *VHL* (64%), *PBRM1* (36%), *SETD2* (20%), and *BAP1* (13%) [10]. Moreover, while *VHL*, *PBRM1*, *SETD2*, and *BAP1* mutations are the most dominant in ccRCC, mutations of *MET*, *SETD2*, *NF2*, *TP53*, and *PTEN* are more common in papillary RCC and chromophobe RCC, respectively [17].

In this study, we demonstrated that the most common pathogenic/likely pathogenic gene alterations in a small mixed RCC cohort were found in *CHEK2*, *VHL*, *PBRM1*, *SETD2*, and *BRCA2*. In addition, we discovered an association between longer TFFS in patients with *SETD2* gene alterations and shorter TFFS in patients with *BRCA2* mutations.

*SETD2* is a methyltransferase responsible for the trimethylation of lysine 36 on histone 3 (H3K36me3), which is essential for the transcription of genes in the metabolic pathway and DNA damage repair, which is regulated by the VEGF/PDGF pathway [10]. Reports described the role of mutated SET2D in chemotherapy resistance [18, 19]. Moreover, changes in SETD2 levels have been linked to resistance to the TKI imatinib [20]. The CAB inhibits the downstream signaling of the VEGF/PDGF pathway by interfering tyrosine kinase receptors [10]. Additionally, *SETD2* alterations decrease the methylation of Histone 3. Both result in a lower transcription of genes of the DNA repair pathway as well as cell proliferation and, therefore, in consecutive cell death [10, 21]. However, none of the proven pathogenic/likely pathogenic gene alterations was associated with OS.

The protein expression of *SETD2* is associated with better cancer-specific survival in mRCC [22]. Chen et al. observed an objective response in a mixed non-ccRCC cohort treated with Nivolumab and CAB in papillary RCC 5/6 patients with NF2 or FH gene mutation. Only 1 of 6 patients with papillary RCC presented treatment response by the combination therapy. Also, commonly mutated genes for patients with chromophobe RCC were *TP53* and *PTEN*. The authors concluded from small cohort data that genomic alterations may be associated with different treatment responses in RCC [23]. For the treatment with the TKI Sunitinib, alterations in the genes *G6PD*, *CRP1B*, *SETD2*, *TET2*, *SYNE1*, and *DCC* seem predictive for treatment response [24].

The limitation of our study is the small number of patients included. Moreover, patients in different treatment lines of CAB and with different RCC subtypes were investigated. Due to the heterogeneity of the patient cohort, it is difficult to make generalized statements on the influence of the discovered association of gene alterations to the treatment response. However, despite this inhomogeneity, our data reveal that testing of genomic alteration might impact treatment response. Our data need to be further investigated in a homogenous and prospective cohort of patients treated by 2L CAB after ICP/ICP or ICP/TKI failure in patients with m RCC.

## Conclusions

Pathogenic variant genes may indicate treatment response to systemic therapy in mRCC. Patients with alterations of the *SETD2* gene show a longer response to CAB. Therefore, adapting this novel molecular marker to existing risk scores might be a promising option to predict treatment response in mRCC.

## Supplementary Information

Below is the link to the electronic supplementary material.

Supplementary file1 (DOCX 16 KB) Supplement Table 1: Patient’s characteristics

Supplementary file2 (DOCX 13 KB) Supplement Table 2: Variant assessment of genomic alterations

Supplementary file3 (DOCX 15 KB) Supplement Table 3: Treatment response and duration of treatment response with Cabozantinib in 1L and ≥ 2L
