# Supplementary material for: Identification of genomic drivers for the therapeutic response of Cabozantinib in patients with metastatic renal cell carcinoma
Source: World J Urol. 2024 Feb 22;42(1):94. doi: 10.1007/s00345-024-04783-y (PMC10884127; doi:10.1007/s00345-024-04783-y)
Supplement: Supplementary file 1 — Supplementary file1 (DOCX 16 KB) Supplement Table 1: Patient’s characteristics [file 345_2024_4783_MOESM1_ESM.docx]

| **Variable** |  |
| --- | --- |
| age at diagnosis (years, median, interquartile range (IQR)) | 59 (42-83) |
| Appearance of metastasis (n (%))   - synchronous - metachronous | 6  13 |
| IMDC risk group at time point of primary diagnosis of mRCC   - favourable risk (n) - intermediate risk (n) - poor risk (n) | 6 (46)  6 (48)  1 (8) |
| Median number of systemic treatment (n; range) | 2 (1-5) |
| Staging of primary tumor (n=15) (n (%))   - pT ≥ 3 - pN1 - L1 - V1 | 12 (80)  3 (20)  2 (13)  6 (40) |
| Histopathology of the primary cancer (n (%))   - clearcell RCC - chromophobe RCC - papillary RCC - Dedifferentiated/chromophobe | 11 (61)  2 (11)  1 (6)  3 (16) |
| Primary treatment (n (%))   - Surgical Local treatment (kidney) - Surgical local treatment (kidney + metastasis) - Systemic treatment | 12 (63)  2 (10)  5 (26) |
