# Supplementary material for: Identification of genomic drivers for the therapeutic response of Cabozantinib in patients with metastatic renal cell carcinoma
Source: World J Urol. 2024 Feb 22;42(1):94. doi: 10.1007/s00345-024-04783-y (PMC10884127; doi:10.1007/s00345-024-04783-y)
Supplement: Supplementary file 2 — Supplementary file2 (DOCX 13 KB) Supplement Table 2: Variant assessment of genomic alterations [file 345_2024_4783_MOESM2_ESM.docx]

| **Pathogenic** | **likely pathogenic** | **uncertain significance*** | **likely benign/benign*** |
| --- | --- | --- | --- |
| ARID1A (1) | BRAF (1) | ALK | ARAF |
| ASXL1 (1) | BRCA2 (2) | APC | ARID1B |
| BAP1 (1) | CDKN2A (1) | AR | ARID2 |
| KDM5C (1) | **CHEK2 (14)** | ARID2 | ATM |
| KMT2C (2) | EPAS1 (2) | AKRKC | ATR |
| MTOR (1) | KDM5C (1) | BAP1 | ATRX |
| NF2 (1) | NF1 (1) | BCOR | AXIN1 |
| NOTCH1 (1) | PTEN (1) | FANCC | AXIN2 |
| NOTCH2 (2) | TSHR (1) | FANCD2 | BARD1 |
| **PBRM1 (7)** | VHL (3) | FGFR4 | BIRC3 |
| PPM1D (1) |  | MDM4 | BRCA2 |
| **SET2D (7)** |  | NOTCH2 | CDKN1B |
| SMARC B1 (1) |  | PPRM1 | EXO1 |
| SUZ12 (1) |  | PMS2 | GRIN2A |
| TP53 (2) |  | RAD51C | KMT2C |
| **VHL (11)** |  | SDHA | SETBP1 |
|  |  | ZNF217 | ZNF217 |

(n=alterated allels)
