# Supplementary material for: Identification of genomic drivers for the therapeutic response of Cabozantinib in patients with metastatic renal cell carcinoma
Source: World J Urol. 2024 Feb 22;42(1):94. doi: 10.1007/s00345-024-04783-y (PMC10884127; doi:10.1007/s00345-024-04783-y)
Supplement: Supplementary file 3 — Supplementary file3 (DOCX 15 KB) Supplement Table 3: Treatment response and duration of treatment response with Cabozantinib in 1L and ≥ 2L [file 345_2024_4783_MOESM3_ESM.docx]

Suppl. Table 2

| 1L systemic treatment   - Cabozantinib - Sunitinib - Pazopanib - IO/TKI - Ipilimumab/Nivolumab | (n (best treatment response))  7 (SD: n=3, PR: n=3)  6 (SD: n=2; PR: n=2, PD: n=2)  2 (PR: n=2)  2 (PR: n=1; PD: n=1)  1 (SD) | Median TFFS (months; IQR)  9 (2-26)  5.5 (2.5-22)  12 (11-12)  4.5 (3-4.5)  2 (-) |  |
| --- | --- | --- | --- |
| Number of treatment line with Cabozantinib   - 1L - 2L - 3L - 4L - Best treatment response (n(%))   - SD   - PR - Median TFFS (months; IQR) | 7 (SD: n=3, PR: n=3)  6 (SD: n=5, PR: n=1)  5 (SD: n=5)  1 (SD)  14 (78)  4 (22)  10.4 (5.2-26) | Median TFFS (months; IQR)  9 (IQR 2-26)  11.5 (IQR 7.3-20)  12 (IQR 3-4)  10 | Median OS after beginning Cabozantinib (months; IQR)  21.95 (IQR 2.3-25.8)  20.5 (IQR 15.4-28.6)  14.9 (IQR 11.5-44.21)  22.24 |
